# Neural cell-surface and intracellular autoantibodies in patients with cognitive impairment from a memory clinic cohort

**DOI:** 10.1007/s00702-021-02316-0

**Published:** 2021-03-06

**Authors:** Niels Hansen, Berend Malchow, Inga Zerr, Winfried Stöcker, Jens Wiltfang, Charles Timäus

**Affiliations:** 1 Department of Psychiatry and Psychotherapy, University Medical Center of Göttingen, University of Goettingen, Von-Siebold-Str. 5, 37075 Goettingen, Germany; 2 Department of Neurology, University of Goettingen, Robert-Koch Str. 40, 37075 Goettingen, Germany; 3 German Center for Neurodegenerative Diseases (DZNE), Von-Siebold-Str. 3a, 37075 Goettingen, Germany; 4 Neurosciences and Signaling Group, Institute of Biomedicine (iBiMED), Department of Medical Sciences, University of Aveiro, Aveiro, Portugal; 5 Euroimmun Reference Laboratory, Seekamp 31, 23650 Luebeck, Germany

**Keywords:** Autoimmunity, Cognitive impairment, Neural autoantibodies

## Abstract

Autoantibody-associated cognitive impairment is an expanding field in geriatric psychiatry. We aim to assess the association between the presence of specific neural autoantibodies and cognitive performance in a memory clinic cohort. 154 patients with cognitive impairment were included between 2019 and 2020 presenting initially in a memory clinic. We evaluated their patient files retrospectively applying epidemiologic parameters, psychopathology, neuropsychology, intracellular and membrane-surface autoantibodies in serum and cerebrospinal fluid (CSF) and markers of neurodegeneration in CSF. In 26 of 154 patients, we searched for neural autoantibodies due to indicators for autoimmunity. In 15/26 (58%) of patients we detected serum and/or CSF autoantibodies. We identified autoantibodies against intracellular or cell-surface antigens in 7 of all 26 (27%) patients with cognitive dysfunction, although we cannot exclude patients with potential specific autoantibodies lacking autoimmune indicators. There were no significant differences between psychopathological and neuropsychological profiles in groups of patients with cognitive impairment comprising patients with autoantibodies (ABS + COG), no autoantibodies (ABS − COG), and Alzheimer’s disease (ADCOG). Concerning our CSF parameters, we detected intrathecal IgG synthesis in 14% of ABS + COG and in 13% of ABS − COG patients, whereas no intrathecal IgG synthesis was found in ADCOG patients. Furthermore, CSF Aß42 was significantly diminished in the ADCOG compared to the ABS + COG group (*p* < 0.05). In addition, the Aß42/40 ratio was lower in ADCOG patients than in the ABS + COG or ABS − COG group (*p* < 0.05). Our findings reveal the underestimated occurrence and autoantibodies’ potential role in patients presenting cognitive impairment. Furthermore, the patients with possible Alzheimer’s disease might be differentiated from autoantibody-positive patients via a reduced Aß42 and Aß42/40 ratio in the CSF. The antibody-type varies between patients to a relevant degree, thus demonstrating the need for more research to identify subgroup-specific phenotypes. These pilot study results open an avenue for improving diagnosis and treatment in a memory clinic.

## Introduction

Autoantibody-based clinical neurological and psychiatric syndromes are a rapidly growing field in clinical neuropsychiatry and geriatric psychiatry as they often present with cognitive decline as the first or concomitant symptoms (Gibson et al. 2020; Bartels et al. 2019; Arino et al. 2016; Loane et al. 2019; Sechi and Flanagan 2019). A recent study focusing on the increasing frequency of *N*-methyl-d-aspartate receptor antibodies (NMDAR)-mediated atypical dementia (Gibson et al. 2020). In this context, atypical dementia entailing an early-onset and atypical presentation is more often reported to be associated with NMDAR autoantibodies (Gibson et al. 2020). Individual specific autoantibodies such as α-amino-3-hydroxy-5-methyl-4-isoxazolepropionic acid receptor (AMPAR) subunit ionotropic glutamate receptor 3 (GluA3) autoantibodies (Palese et al. 2020) or contactin-associated protein-like 2 (CASPR2) autoantibodies (Guo et al. 2020) have been demonstrated in homogeneous groups of patients with cognitive impairment. They reported a high number of cancer patients with specific autoantibodies (22.3%) with and without cognitive impairment (Bartels et al. 2019). Some patients in this cohort suffering cognitive impairment (36.9%) had autoantibodies against NMDAR, myelin oligodendrocyte glycoprotein (MOG), pre glycine receptor alpha 1 (pre-GLRA1), glutamic acid decarboxylase 65 (GAD65), Rho GTPase-activating protein 26 (ARHGAP26) and Hu antigen (Bartels et al. 2019) suggesting a manifold illustration of specific neural autoantibodies existing in patients with cognitive impairment. No study to date has addressed the frequency of a broad spectrum of serum and CSF neural cell-surface and intracellular autoantibodies in patients with cognitive impairment in a memory clinic cohort. There have been studies addressing a wide, but different spectrum of autoantibodies than those reported to be associated with Alzheimer´s disease, such as autoantibodies against the 5-hydroxytryoptamine receptor, dopamine receptor and glutamate receptors such as NMDAR (Wu et al. 2016), anti-ganglioside or anti-adenosine triphosphate synthase antibodies (Colasanti et al. 2010), or nucleosome assembly protein 1-like 3 and microtubule-associated protein 4 autoantibodies (Wang et al. 2020). We thus investigated the occurrence of various autoantibodies against cell-surface and intracellular antigens that have been only partly reported to date in patients with cognitive impairment from a memory clinic sample. Screening for autoantibodies against intracellular antigens is highly relevant diagnostic approach and therapeutic indication, as these autoantibodies are often involved in tumor immunity.

## Materials and methods

### Patients and procedures for autoantibody search

We consecutively enrolled a sample of 154 patients in one year between 2019 and 2020 in our retrospective and observational study. What these patients had in common was their initial presentation to a physician due to cognitive dysfunction in the memory out- and inpatient unit in the Department of Psychiatry and Psychotherapy, University Medical Center of Göttingen. We looked for autoantibodies in serum (*n* = 26) or cerebrospinal fluid (CSF) (*n* = 22) in 26 of these 154 patients (Table 1) who presented additional clinical features indicating possible underlying autoimmunity such as the “yellow flags” or “red flags” described previously (Herken and Prüss 2017). The occurrence of one “yellow flag” or “red flag” sufficed for us to look for additional specific autoantibodies in either serum or/and CSF in 26 patients. We did not search for autoantibodies in 128 patients with cognitive impairment ranging from subjective cognitive decline (SCD) to mild cognitive impairment (MCI) and dementia. Our study was conducted in accordance with the Declaration of Helsinki, and we received ethical approval for our retrospective study from our local ethics committee.

**Table 1:** Demographics, psychopathology, neuropsychological and laboratory parameters of patients groups

	ABS + COG	ABS − COG	ADCOG	Statistics
	*N* = 14	*N* = 8	*N* = 9	*p*-value
Demographics				
Age (years)	68.6 ± 3.5	65.4 ± 3.5	72 ± 3.8	0.484
Sex (*n*, female)	7	5	6	0.457
Duration of symptoms (years)	1.8 ± 0.6	1.7 ± 0.5	3.4 ± 1.3	0.316
Psychopathology				
Disorientation score 0–1, (% of patients)	0.7 ± 0.1 (64)	0.4 ± 0.2 (38)	0.55 ± 0.2 (56)	0.906
Depression score 0–1, (% of patients)	0.6 ± 0.1 (57)	0.9 ± 0.1 (88)	0.77 ± 0.2 (77)	0.538
Suicidality score 0–1, (% of patients)	0.2 ± 0.9 (14)	0.25 ± 0.2 (25)	0 (0)	0.127
Anxiety score 0–1, (% of patients)	0.5 ± 0.1 (43)	0.62 ± 0.2 (63)	0.44 ± 0.16 (44)	0.854
OCB score 0–1, (% of patients)	0.07 ± 0.07 (7)	0 (0)	0 (0)	0.434
Delusions score 0–1, (% of patients)	0.14 ± 0.09 (14)	0.13 ± 0.11 (13)	0 (0)	0.290
Hallucinations score 0–1, (% of patients)	0.07 ± 0.06 (7)	0.13 ± 0.11 (13)	0 (0)	0.284
Aggression score 0–1, (% of patients)	0.3 ± 0.11 (28)	0.25 ± 0.15 (25)	0 (0)	0.519
Apathy score 0–1, (% of patients)	0.08 ± 0.06 (7)	0.13 ± 0.12 (13)	0 (0)	0.627
Sleep dysfunc. score 0–1, (% of patients)	0.61 ± 0.1 (57)	0.42 ± 0.17 (38)	0.33 ± 0.15 (33)	0.171
Yellow flags score 0–1, (% of patients)	0.07 ± 0.06 (7)	0.38 ± 0.17 (38)	0 (0)	–
Red flags score 0–1, (% of patients)	0.92 ± 0.06 (93)	0.75 ± 0.15 (75)	0.55 ± 0.16 (56)	0.572
Geriatric depression score	5.5 ± 0.8	8.6 ± 1.6	5.5 ± 4.3	0.404
MRI				
Pathological temporal score	0.28 ± 0.1 (29)	0.42 ± 0.2 (38)	0.57 ± 16 (44)	0.575
Pathological extratemporal score	0.64 ± 0.1 (64)	0.42 ± 0.2 (38)	0.42 ± 15 (33)	0.368
Serum				
CRP mg/L (pathological > 5 mg/l)	8.3 ± 3.2	2.7 ± 0.4	5.6 ± 2.9	0.756
Leukocytes 10^3^ µL (Reference 4–11^3^ µL)	7.2 ± 0.7	5.7 ± 0.6	7.7 ± 0.3	0.198
CSF				
Cell count/µL (pathological: > 5 µL)	1.2 ± 0.6	2.5 ± 1.3	0.6 ± 0.35	˂ 0.05
Lymphocytes in %	73 ± 5.4	87 ± 3.5	76 ± 3.9	0.209
Monocytes in %	27 ± 5.9	12.8 ± 3.5	22 ± 4.3	0.460
Whole protein mg/L	447.4 ± 34.9	472 ± 55	389.3 ± 27	0.126
Albumin mg/L	298.4 ± 27.1	317 ± 45	241.8 ± 21	0.165
IgG mg/L	35.6 ± 3.7	40.6 ± 3.8	23.5 ± 2.9	0.070
IgA mg/L	4.2 ± 0.86	3.8 ± 1.2	3.0 ± 0.6	0.647
CSF				
IgM mg/L	0.59 ± 0.06	0.88 ± 0.3	0.29 ± 0.05	0.062
QAlb %	7.04 ± 0.58	7.5 ± 1.2	5.9 ± 0.53	0.167
QIgG %	3.7 ± 0.41	3.9 ± 0.6	2.8 ± 0.31	0.221
QIgM %	0.81 ± 0.16	0.89 ± 0.21	0.45 ± 0.09	0.182
Lactat mmol/L	1.78 ± 0.06	1.6 ± 0.07	1.6 ± 0.06	0.751
Intrathecal IgG score 0–1 (% of patients)	0.16 ± 0.09 (14)	0.2 ± 0.11 (13)	0 (0)	0.520
Neuropsychological testing				
Semantic fluency (*z*-value)	− 1.1 ± 0.22	0.1 ± 0.7	− 0.55 ± 0.46	0.632
Phonematic fluency (*z*-value)	− 0.85 ± 0.36	− 0.16 ± 0.36	− 0.21 ± 0.48	0.481
Boston naming test	− 1.01 ± 0.34	0.37 ± 0.28	− 0.55 ± 0.57	0.679
MMSE	24.6 ± 1.5	26.6 ± 0.79	22.1 ± 1.7	0.235
MMSE (*z*-value)	− 2.8 ± 0.68	− 1.97 ± 0.62	− 3.3 ± 0.6	0.330
Verbal learning (*z*-value)	− 2.2 ± 0.5	− 1.75 ± 0.58	− 3.43 ± 0.89	0.139
Verbal recall (*z*-value)	− 1.9 ± 0.3	− 1.69 ± 0.49	− 2.26 ± 0.51	0.236
Word intrusions (*z*-value)	− 0.7 ± 0.32	− 0.34 ± 0.45	− 0.38 ± 0.35	0.665
Saving words (*z*-value)	− 2.01 ± 0.31	− 1.08 ± 0.93	− 0.57 ± 0.51	0.966
Discriminability (*z*-value)	− 1.57 ± 0.38	− 1.31 ± 0.44	− 1.95 ± 0.69	0.833
Visuoconstruction (*z*-value)	− 0.93 ± 0.35	− 0.54 ± 0.63	− 0.72 ± 0.58	0.760
Figure recall (*z*-value)	− 1.94 ± 0.36	− 2.7 ± 0.52	− 1.9 ± 0.56	0.602
Saving figures (*z*-value)	− 1.75 ± 0.29	− 1.6 ± 0.7	− 1.05 ± 0.65	0.919
Trail making test A (*z*-value)	− 0.87 ± 0.27	− 0.44 ± 0.51	− 0.36 ± 0.4	0.844

*ABS + COG *patients with autoantibodies and cognitive impairment, *ABS – COG *patients with cognitive impairment but no autoantibodies, *ADCOG *Alzheimer disease patients with cognitive impairment, *CRP *c-reactive protein, *dysf *dysfunction, *IgG *immunoglobulin G, *IgM *immunoglobulin M, *MMSE *Mini mental status examination, *OCB *oligoclonal bands, *QAlb *quotient Albumin, *QIgG *quotient immunoglobulin G, *QIgM *quotient immunoglobulin M

### Assessing cognition

We applied the Consortium to Establish a Registry for Alzheimer’s Disease (CERAD) plus testing to assess cognitive function. Patients’ cognitive impairment was classified by their CERAD assessment results. A patient’s cognitive impairment was termed SCD if the subject reported cognitive dysfunction, but the neuropsychological performance on CERAD ranges above − 1.5 of the standard deviation (SD) on each subtest of the CERAD test armamentarium considering data normalized for age, education, and sex (Jessen et al. 2018). Cognitive performance was classified as MCI if the subject reported cognitive decline not affecting daily living abilities in line with the Jessen criteria (Jessen et al. 2018; Petersen et al. 2014) and the CERAD testing result fell below − 1.5 SD in the delayed recall subtest of the CERAD word list in respect to age, education, and sex-adapted normative data, indicating impaired episodic memory.

### Assessing psychopathology

Psychopathology was classified by relying on patients’ self-reports and relatives´ statements from the patient letters assessing these terms: orientation, depression, anxiety, obsessive–compulsive behaviour, delusions, hallucinations, aggression, apathy, sleep dysfunction, eating abnormalities or libido. Each item was evaluated as either affected (score = 1) or unaffected (score = 0). Psychopathology profiles were evaluated retrospectively from patient records. We also applied the geriatric depression scale (Brink et al. 1982) to depict the severity of a depressive syndrome in elderly patients (GDS, 0–5 = no depression, 5–10 = mild depression, > 10 = severe depression) before specific autoantibody testing.

### Group classification

We formed three different groups of patients from our population of 154 patients. (1) From our group of patients tested for autoantibodies (*n* = 26), we formed a group of 14 patients with cognitive impairment (*n* = 8 dementia, *n* = 5 MCI, *n* = 1 SCD) and proof of specific autoantibodies in either serum or CSF, calling them the “ABS + COG” group. (2) Another group of 8 patients in whom we sought autoantibodies but detected no specific autoantibodies in serum or CSF revealed cognitive impairment—we refer to them as the “ABS − COG” group (ABS − COG: *n* = 2 dementia, *n* = 6 MCI). (3) The third group called the “ADCOG” group (6 patients with dementia and 2 patients with MCI) served as our disease control-group consisting of 9 patients from our 154 patients with possible Alzheimer’s disease (AD) due to either a typical clinical presentation according to the McKhann et al. (2011) clinical criteria in conjunction with a temporomesial atrophy pattern in MRI and/or pathological levels of molecular CSF biomarkers suggesting AD concurring with Jack et al. (2018). In these 9 patients, we tested for 4 autoantibodies due to the presence of at least one “red flag” or “yellow flag” according to Herken and Prüss (Herken and Prüss 2017). Thus, it is important to mention that we did not classify our groups by relying on the presence of “red flags” or “yellow flags”. No specific autoantibodies were detected in 3 of 9 ADCOG-group patients (33%). Anti-myelin autoantibodies were identified in one patient presenting a molecular biomarker signature suggesting Alzheimer´s disease, and myelin antibodies have been reported to be associated with Alzheimer´s disease (Papuc et al. 2015), thus we added that patient to our “ADCOG” group.

### Criteria for clinical diagnosis

To diagnose Lewy body dementia (LBD), we applied the McKeith criteria (2017), for AD we utilized the McKhann criteria (2011), for vascular dementia (VaD) and mixed dementia (MD) we used the Gorelick et al. criteria (2011) including possible VaD and MD, for Parkinson’s disease dementia (PDD) we utilized the Goetz et al. criteria (2008) and to diagnose frontotemporal dementia (FTD) we exerted the Gorno Tempini et al. criteria (2011). We applied the Graus criteria to diagnose possible autoimmune encephalitis according to international guidelines (Graus et al. 2016) encompassing a subacute onset of (1) short-term memory abnormalities, psychiatric or mental symptoms, (2) the existence of another item (focal neurological deficit, seizures, CSF pleocytosis or MRI typical for encephalitis) and (3) the exclusion of alternative reasons for that clinical symptomatology.

### Autoantibody analysis

The specific autoantibodies analyzed in the Euroimmun reference laboratory (Lübeck) were: antibodies against GAD65, Zic4, DNER/Tr, ITPR1 = Inositol 1, 4, 5-triphosphate receptor 1, Recoverin, SOX1, Ma2, Amphiphysin, CV2, Ri, Yo, HuD via immune line blots. We also sought autoantibodies against the following neural antigens via recombinant cell-based fluorescent technique: NMDAR, leucine-rich glioma inactivated protein 1 (LGI1), Glycin, AMPAR1/2, γ-amino butyric acid A/B receptor (GABAAB1/2), dipeptidyl-peptidase-like protein 6 (DPPX), CASPR2, potassium voltage-gated channel subfamily A member 2 (KCNA2), IgLON5, MOG, and Aquaporin4.

### Molecular markers

We considered a biomarker result as positive suggesting AD if it showed either a reduced ß-amyloid 1–42 (Aß42) or reduced ß-amyloid 1–42/1–40 ratio (Aß-ratio) and elevated a phosphorylated tau 181 (pTau181) or tau protein in CSF. In one patient no CSF was investigated, but ß-amyloid-positron emission tomography (322 MBq Fluor-18-Florbetaben, Department of Nuclear Medicine, University Medical Center Göttingen) showed an elevated brain amyloid-load. We relied on the following reference values for our neurodegenerative biomarkers provided by the neurochemical CSF laboratory in the Göttingen University Neurological Department (tau protein: pathological > 450 pg/ml, p-Tau 181 protein: pathological > 61 pg/ml, Aß42 pathological: ˂ 450 pg/ml and Aß ratio: pathological ˂ 0.5). In addition, we enlisted the Laboratory of Clinical Neurochemistry and Neurochemical Dementia Diagnostics at University Hospital Nürnberg Erlangen to determine the Aß ratio (Aß ratio: pathological ˂ 0.5).

### Laboratory investigations

Other blood parameters for determination of peripheral inflammation such as C-reactive protein (CRP) and leukocytes were assessed in the University Medical Center’s interdisciplinary laboratory in Göttingen. The CSF parameters immunoglobulin G (IgG), immunoglobulin A (IgGA), immunoglobulin M (IgM), albumin, and intrathecal IgG synthesis were assessed in the neurochemistry CSF laboratory in the Department of Neurology’s in University Medical Center in Göttingen.

### Neuroimaging

Temporal and extratemporal magnetic resonance imaging (MRI) were performed either in the University Medical Center Göttingen’s Neuroradiology Department with a 1.5 T MRI (Siemens AvantoFit) or off-site in a neuroradiologic center in Göttingen. We developed a score describing the degree of affection of the temporal and extratemporal brain regions (Score = 0 means unaffected, score 1 means affected). Furthermore, we documented MRI abnormalities between groups.

### Statistics

Descriptive statistics were used to analyze the frequency of autoantibodies and other demographic parameters in our cohort. ANOVA was used to compare age in years, duration of symptoms in years, sex, psychopathology scores (0–1), GDS values, MMSE values, MRI scores (0–1), CSF parameters [(intrathecal IgG synthesis (score = 0 not present, score 1 = present)] and CSF neurodegenerative markers between groups (ABS + COG, ABS − COG- and ADCOG group). Furthermore, LSD post hoc tests with Bonferroni correction were used for evaluate differences between groups (ABS + COG, ABS − COG- and ADCOG group). Mann–Whitney *U*-tests were utilized to compare patients with different MCI etiologies to those with diverse dementia etiologies. A *p*-level of *p *˂ 0.05 was considered significant.

## Results

### Basic memory clinic population

**Fig. 1:**
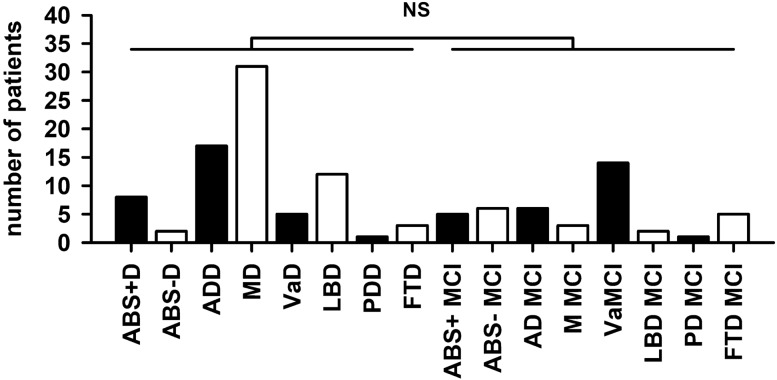
Memory clinic patient cohort. We detected no significant differences between the numbers of patients with different diagnoses and those with dementia and those with mild cognitive impairment (MCI). *ABS + D *autoantibody-positive with dementia, *ABS-D *autoantibody-negative with dementia, *AD *Alzheimer’s disease, *MD *mixed dementia, *VaD *vascular dementia, *LBD *Lewy body dementia, *PDD *Parkinson’s disease dementia, *FTD *frontotemporal dementia, *ABS + MCI *autoantibody-positive patients with mild cognitive impairment, *ABS-MCI *autoantibody-negative patients without mild cognitive impairment, *AD MCI *Mild cognitive impairment with Alzheimer etiology, *M MCI *mixed etiology of mild cognitive impairment, *NS* non significant, *VaMCI *vascular disease with mild cognitive impairment, *LBD MCI *Lewy body disease with mild cognitive impairment, *PD MCI *Parkinson’s disease with mild cognitive impairment, *FTD MCI *frontotemporal disease with mild cognitive impairment

The symptoms of cognitive dysfunction in 154 patients were classified as subjective cognitive decline (SCD), mild cognitive impairment (MCI) or dementia according to the aforementioned criteria. The number of patients with dementia with diverse diagnoses does not differ from that of patients with MCI due to diverse diagnoses (Fig. 1, Mann–Whitney *U*-test *p* = 0.51). We conducted no specific autoantibody tests in 128 patients: these patients suffered cognitive impairment due to the following suspected diagnoses: vascular disease (VaD) (*n* = 1 SCD, *n* = 14 MCI (VaMCI) and *n* = 5 dementia), neurodegenerative disease [AD: *n* = 5 MCI (MCI with Alzheimer’s disease etiology), *n* = 14 dementia (ADD); LBD: *n* = 2 MCI, *n* = 12 dementia; PDD: *n* = 1 MCI, *n* = 1 dementia; FTD: *n* = 5 MCI, *n* = 3 dementia], mixed etiology (*n* = 3 MCI, *n* = 31 dementia) or other etiologies (*n* = 4 SCD, *n* = 24 MCI and *n* = 3 with dementia). All patients are depicted cross-sectionally; no follow-ups have occurred so far.

### Clinical, psychopathological, neuropsychological characteristics of groups

Sex, age, and years of disease duration did not differ between patient groups (ABS + COG, ABS − COG, ADCOG, Table 1). We detected no differences between groups in their psychiatric presentation regarding their disorientation, obsessive–compulsive behaviour, depression, anxiety, delusions, hallucinations, suicidality, aggression, apathy, sleep, eating abnormalities and libido score (ANOVA, n.s., Table 1). Neuropsychological functions assessed by the CERAD Plus instrument (semantic and phonematic word fluency, learning and consolidation of verbal and figural material, psychomotoric processing speed as well as visuoconstruction, naming) did not differ in their *z*-values between groups in any cognitive subdomain (ANOVA: *F* 13.3, *p* = 0.21, Table 1). For other clinical characteristics as comorbidities see Tables 1 and 2.

**Table 2:** Antibody characteristics, drugs, and comorbidities of patient groups

	ABS + COG *N* (%)	ABS − COG *N* (%)	ADCOG *N* (%)
Cell-surface autoantibody			
CASPR2 Serum	1 (7.1%)	–	–
Glycin Serum	2 (14.3%)	–	–
IgLON5 CSF	1 (7.1%)	–	–
IgLON5 Serum	1 (7.1%)	–	–
KCNA2 Serum	1 (7.1%)	–	–
Myelin Serum	–	–	1 (7.1%)
MOG CSF	1 (7.1%)	–	–
Intracellular antibody			
CV2 Serum	1 (7.1%)	–	–
ITPR1 Serum	1 (7.1%)	–	–
Recoverin Serum	2 (14.3%)	–	–
Titin Serum	1 (7.1%)	–	–
Yo CSF	1 (7.1%)	–	–
Yo Serum	2 (7.1%)	–	–
Unspecific neuropil binding			
CSF Hipp, Thalamus, Cortex	1 (7.1%)	–	–
Serum Hipp, Thalamus, Cortex	1 (7.1%)	–	–
Psychiatric comorbidity			
Agoraphobia − panic disorder	1 (7.1%)	0 (0%)	0 (0%)
Agoraphobia + panic disorder	1 (7.1%)	(0%)	0 (0%)
Bipolar disorder	1 (7.1%)	2 (25%)	0 (0%)
Cyclothymia	1 (7.1%)	0 (0%)	0 (0%)
Major depressive episode	3 (38.5%)	2 (25%)	2 (22%)
Minor depressive episode	2 (14.3%)	1 (12.5%)	1 (11%)
Nicotine dependency	1 (7.1%)	0 (0%)	0 (0%)
Obsessive–compulsive disorder	1 (7.1%)	0 (0%)	0 (0%)
Posttraumatic stress disorder	0 (0%)	1 (12.5%)	0 (0%)
Somatoform autonomic disorder	1 (7.1%)	0 (0%)	0 (0%)
Neurologic comorbidity			
Atypical restless leg syndrome	1 (7.1%)	0 (0%)	0 (0%)
Cerebral ischemic attacks	1 (7.1%)	0 (0%)	0 (0%)
Disc prolapse	0 (0%)	2 (25%)	0 (0%)
Meningioma	1 (7.1%)	0 (0%)	0 (0%)
Migraine	0 (0%)	1 (12.5%)	0 (0%)
Major cerebrovascular disease	0 (0%)	0 (0%)	1 (11%)
Post herpes encephalitis	0 (0%)	1 (12.5%)	0 (0%)
Neuropathy	1 (7.1%)	0 (0%)	1 (11%)
Spinal canal stenosis	0 (0%)	0 (0%)	1 (11%)
Structural epilepsy	1 (7.1%)	1 (12.5%)	0 (0%)
Tension-type headache	2 (14.3%)	0 (0%)	0 (0%)
Temporal lobe epilepsy	2 (14.3%)	0 (0%)	0 (0%)
Tremor	1 (7.1%)	0 (0%)	0 (0%)
Immunologic or autoimmune comorbidity			
Autoimmunthyreoiditis	0 (0%)	1 (12.5%)	0 (0%)
Colitis ulcerosa	0 (0%)	1 (12.5%)	0 (0%)
Monoclonal gammopathy	1 (7.1%)	0 (0%)	0 (0%)
IgM Kappa proteinemia	1 (7.1%)	0 (0%)	0 (0%)
Secondary IgG deficiency	1 (7.1%)	0 (0%)	0 (0%)
Psychopharmacologic drugs			
Antidementiva	4 (21%)	0 (0%)	7 (78%)
Antidepressants	8 (57%)	5 (63%)	2 (22%)
Anxiolytics	1 (7.1%)	0 (0%)	0 (0%)
Mood stabilizer	1 (7.1%)	0 (0%)	1 (11%)
Neuroleptics	1 (7.1%)	1 (12.5%)	1 (11%)
Immunotherapy			
Corticosteroids	4 (27%)	1 (12.5%)	0 (0%)
MRI abnormalities			
Enlarged limbic structures	0 (0%)	0 (0%)	0 (0%)
Generalized cortical atrophy	7 (50%)	0 (0%)	2 (22%)
Altered limbic gray matter	3 (21%)	0 (0%)	2 (22%)
Hippocampal sclerosis	3 (21%)	0 (0%)	2 (22%)
Localized brain atrophy	7 (50%)	3 (38%)	3 (33%)
Macroangiopathy	1 (7.1%)	0 (0%)	1 (11%)
Microangiopathy	8 (57%)	3 (38%)	3 (33%)
Ventricle enlargement	3 (21%)	0 (0%)	2 (22%)
Malignancy			
Bladder carcinoma	1 (7.1%)	0 (0%)	0 (0%)
Colon carcinoma	1 (7.1%)	0 (0%)	0 (0%)
Mammary carcinoma	0 (0%)	1 (12.5%)	1 (11%)
Melanoma	0 (0%)	1 (12.5%)	0 (0%)
Thyroid carcinoma	0 (0%)	0 (0%)	1 (11%)

*ABS + COG *patients with autoantibodies and cognitive impairment, *ABS – COG *patients with cognitive impairment but no autoantibodies**,** *ADCOG *Alzheimer disease patients with cognitive impairment, *CASPR2 *contactin-associated protein like 2, *Hipp *hippocampus, *IgM *immunoglobulin M, *ITPR1 *Inositol 1,4,5-triphosphate receptor 1, *MOG *myelin oligodendrocyte glycoprotein, *N *number

### Autoimmune indicators of groups

We determined autoantibodies in 26 patients due their presenting ≥ 1 indicator of autoimmunity [“red flag” or “yellow flag”]. In 12/14 of (86%) ABS + COG group patients, we detected one ≥ “red flag” comprising another autoimmune disorder (*n* = 2), tremor (*n* = 2), paresthesia (*n* = 1), new-onset headache (*n* = 2), focal neurological disease (*n* = 3), severe cognitive dysfunction unexplained by another diagnosis (*n* = 3), autonomic dysfunction (*n* = 2), whereas 2/14 (14%) presented one “yellow flag” (psychomotor symptoms *n* = 2). The ABS-COG group contained 6/8 (75%) of patients with one “red flag” (infectious prodrom with fever *n* = 1, seizures *n* = 2, paresthesia *n* = 1, decreased level of consciousness *n* = 1, new-onset headache *n* = 1) and 3/8 (38%) patients with one “yellow flag” (dynamic course *n* = 1, personality dynamic changes *n* = 1, fluctuating psychopathology *n* = 1). Furthermore, we identified red flags in 5/9 (56%) ADCOG-group patients (focal neurological disease *n* = 2, new-onset headache *n* = 1, severe cognitive dysfunction not attributable to any other diagnosis *n* = 1, tumor *n* = 1).

### Serum and cerebrospinal fluid autoantibodies

We investigated serum and/or CSF autoantibodies in 26 of 154 (17%) of patients (Tables 2, 3). In 15 of 26 (58%) patients, we detected serum (*n* = 15) or CSF (*n* = 4) autoantibodies (Table 1). However, we cannot rule out that patients lacking autoimmune indicators whom we do not screen for autoantibodies might have possessed specific detectable autoantibodies. AD dementia was later assumed in one of those patients with positive autoantibodies (serum myelin antibodies). Our 14 ABS + COG patients did not receive a concomitant cognitive-impairment diagnosis due to major cerebrovascular disease. The specific autoantibodies we detected in 14 patients were: *n* = 1 CASPR2 abs, *n* = 1 CV2, *n* = 3 Recoverin, *n* = 2 KCNA2, *n* = 2 Glycin, *n* = 1 Yo, *n* = 1 ITPR1, *n* = 1 IgLON5 and *n* = 1 Titin in serum as well as *n* = 1 Yo abs, *n* = 1 IgLON5 abs and *n* = 1 MOG abs in CSF. We detected 11 different autoantibodies in serum and CSF in 15 patients with autoantibodies, thereby revealing the heterogeneous spectrum of specific autoantibodies that patients with cognitive impairment may exhibit. Antibodies against cell-surface and intracellular antigens were found in 7/14 (50%) of ABS + COG patients, respectively (see Table 3 for frequencies of specific autoantibodies). Furthermore, we detected unspecific neuropil antibodies in one patient’s cerebrum, thalamus, and in hippocampus in the serum and CSF. In contrast, in 11 of 26 patients (42%, Table 3) we identified no antibodies in either serum or CSF. Possible autoimmune encephalitis was diagnosed in 8 of those 14 patients (57%) with serum and/or CSF autoantibodies and in one patient of those with no serum or CSF autoantibodies (12.5%). We thus diagnosed possible autoimmune encephalitis in 5.8% of the patients in our entire memory clinic sample. Furthermore, 5 of 14 antibody-positive patients (36%) fulfilled the criteria for a probable autoimmune-based cognitive impairment according to Hansen et al. (Hansen et al. 2020a). See Tables 1 and 2 for MRI abnormalities, tumors, and drug history of patient groups.

**Table 3:** Specification of cohort following autoantibody testing

Parameters	*N* (%)
Patients in whom we sought autoantibodies, *N* = 26	
Hit rate for serum and CSF autoantibodies	15 (58)
Hit rate for positive serum autoantibodies	15 (58)
Hit rate for positive CSF autoantibodies	4 (15)
Positive CSF autoantibody result	4 (15)
Positive serum autoantibody result	15 (58)
Cell-surface autoantibodies serum and CSF	7 (27)
Intracellular autoantibodies serum and CSF	7 (27)
Cell-surface autoantibodies serum	6 (23)
Intracellular autoantibodies serum	6 (23)
Cell-surface autoantibodies CSF	2 (8)
Intracellular autoantibody CSF	1 (4)
Negative serum and CSF autoantibody result	11 (42)
ABS + COG patients, *N* = 14	
Cell-surface autoantibodies serum and CSF	7 (50)
Intracellular autoantibodies serum and CSF	7 (50)
Cell-surface autoantibodies serum	6 (43)
Intracellular autoantibodies serum	6 (43)
Cell-surface autoantibodies CSF	2 (14)
Intracellular autoantibody CSF	1 (7)
Unspecific neuropil antibody CSF	1 (7)

*ABS + COG *patients with autoantibodies and cognitive impairment, *CSF *cerebrospinal fluid, *N *number

### Molecular markers of groups

**Fig. 2:**
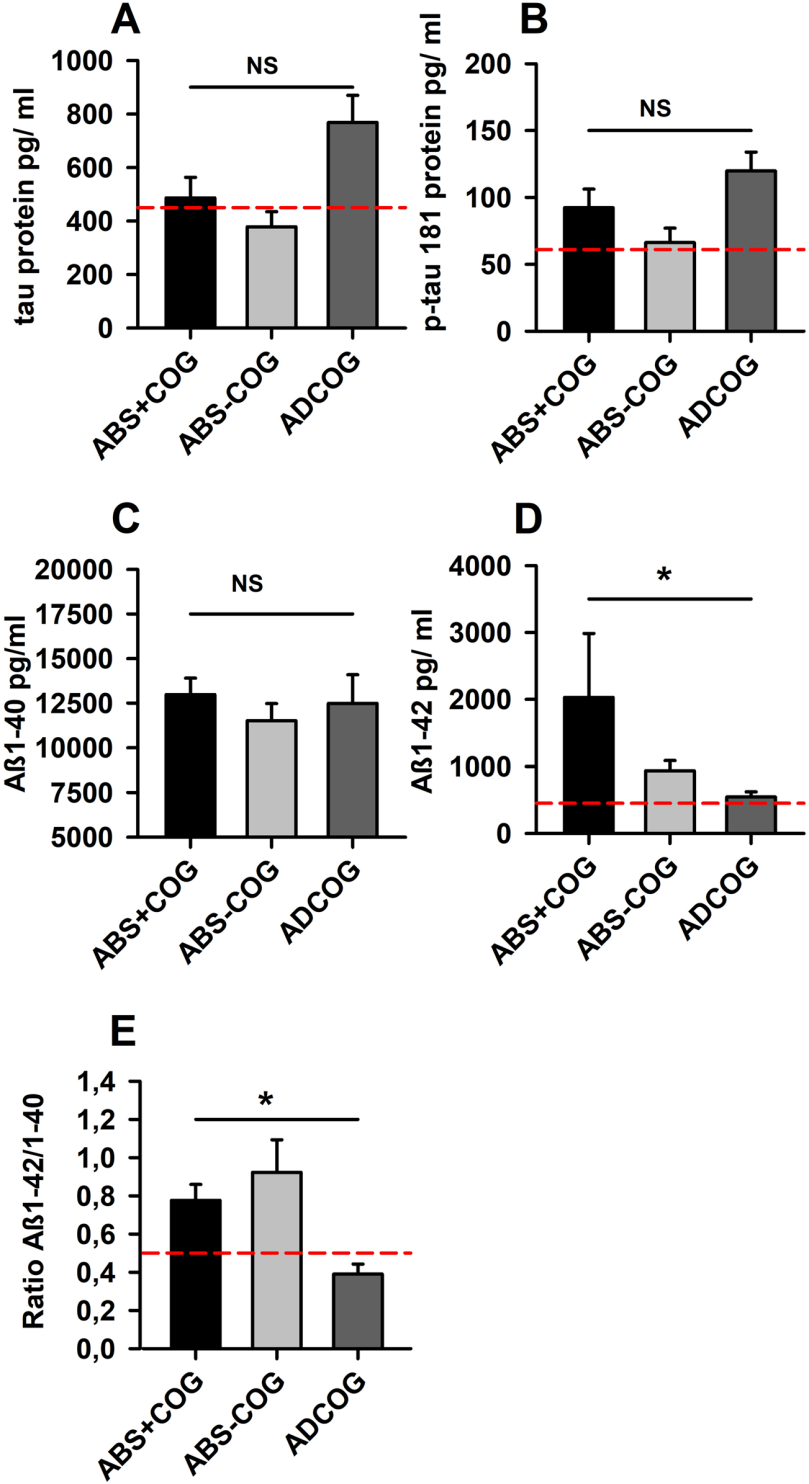
Molecular markers of groups. The Aß1–142/1–40 ratio and Aß1–42 were significantly reduced in Alzheimer´s disease patients with cognitive impairment (ADCOG) when compared with those patients with cognitive impairment and autoantibodies (ABS + COG) as well as without autoantibodies (ABS − COG). The dashed red lines indicate the cut-off laboratory values for each molecular marker. *p < 0.05, ANOVA. *Aß1-40* Beta Amyloid 40, *Aß1-42* Beta Amyloid 42, *NS* non significant, *p-tau 181* phosphorylated tau protein 181

We detected no significant differences in CSF Tau, pTau181, and Aß40 between groups (ABS + COG, ABS − COG, ADCOG). However, a significant difference in the Aß42 CSF content emerged between groups (ABS + COG, ABS − COG, ADCOG) (ANOVA: *F* = 4.06, *p* < 0.05, Fig. 2). CSF Aß42 was significantly more reduced in the ADCOG than the ABS + COG group, confirming one typical positive biomarker for diagnosing AD (Post Hoc LSD test: *p* < 0.05, Fig. 2). In addition, the Aß42/40 ratio was lower in ADCOG patients confirming AD, but not in the ABS + COG or ABS − COG (ANOVA: *F* = 4.06, *p* < 0.05, Fig. 2).

### Laboratory results of groups

CSF data are depicted in Table 1. Intrathecal IgG synthesis was observed in 14% of ABS + COG and in 13% of ABS − COG patients, whereas the ADCOG patients revealed no intrathecal IgG synthesis. CSF cell counts differed significantly between groups (ABS + COG, ABS − COG, ADCOG) (ANOVA: *F* = 4.1, *p* < 0.05), but on average, no pleocytosis was observed in any group (ABS + COG, ABS − COG, ADCOG), and the LSD post hoc tests demonstrated no significant group differences between disease groups (ABS + COG, ABS − COG, ADCOG). Our other CSF data, such as lymphocytes in %, monocytes in %, whole protein, albumin, IgG, IgA, IgM, the quotient of albumin in %, the quotient of IgG, IgM, IgA in %, lactate, and the presence of intrathecal IgG synthesis score did not differ between groups either (ABS + COG, ABS − COG, ADCOG). We observed no correlation between a blood-brain barrier disturbance or the albumin quotient and the level of cognitive impairment and psychopathological profile.

### Immunotherapy

The majority of ABS + COG patients (but only one ABS − COG group patient) underwent immunotherapy. 50% of ABS + COG patients with CSF autoantibodies were given intravenous methylprednisolone (Table 1). The others did not receive immunotherapy for different reasons (no presentation, immunotherapy refused). 6/8 (75%) of patients with autoantibody-positive possible autoimmune encephalitis and 3/5 (60%) patients with probable autoimmune based cognitive impairment received intravenous methlyprednisolone. Only one patient with autoantibody-negative encephalitis underwent methylprednisolone therapy.

## Discussion

Neural cell-surface and intracellular autoantibodies are detected in 15 of 26 (58%) of memory-clinic patients with potential indicators of autoimmunity and cognitive impairment varying from mild cognitive impairment to dementia. We are the first to report the relatively broad and heterogeneous autoantibody spectrum in autoantibody-positive patients presenting 11 specifically detectable autoantibodies and suffering cognitive impairment ranging from MCI to dementia. The high rate of autoantibody detection (58%) in our cohort was due to preselected patients via the presence of possible autoimmune indicators according to the classification of Herken and Prüss (2017). Nevertheless, we do not know the frequency of specific autoantibodies in our 128 patient sample in whom we did not seek antibodies. Our retrospective survey revealed no specific psychopathology and laboratory profile (apart from a reduced Aß42 and Aß42/40 ratio in the ADCOG, but not in the ABS + COG and ABS − COG groups) that clearly differentiates autoantibody-mediated cognitive impairment from biological Alzheimer´s disease.

### Spectrum of specific autoantibodies associated with cognitive impairment

We were unable to confirm Gibson’s findings (2020), namely the higher frequency of NMDAR autoantibodies in patients with atypical dementia in our cohort of autoantibody-positive patients with cognitive dysfunction. Surprisingly, certain autoantibodies have never yet been associated with cognitive impairment, such as the Recoverin antibodies known to mediate autoimmune retinopathy (Oporto Caroca and Oporto Caroca 2019). Frontal atrophy probably due to a neurodegenerative process after an inflammatory state has been described in conjunction with Yo autoantibodies by a study of Endres et al. (2015). Other autoantibodies such as CASPR2, IgLON5, Glycin, and MOG have been reported to be associated with cognitive dysfunction in individual cases and case series (Hansen et al. 2020b; Baba et al. 2019; Swayne et al. 2018; Van Sonderen et al. 2016). In contrast, ITPR1 and KCNA2 autoantibodies have so far not been reported in patients with cognitive dysfunction as the predominant clinical syndrome. The autoantibodies in our cohort patients represent a continuum from possible autoimmune encephalitis to cognitive dysfunction as a clinically-isolated syndrome associated with autoantibodies but not revealing any other evidence of autoimmune encephalitis. The cognitive impairment could be due to an encephalopathy involving functionally disturbed brain function due to interfering synaptic protein autoantibodies such as CASPR2, KCNA2 or glycin autoantibodies in strategically cognition-relevant structures like the hippocampus. Indeed, glycin receptors are expressed in the hippocampus, and are responsible for inhibitory transmission. Glycin autoantibodies in the hippocampus might explain why the memory function in these patients is disturbed, through increased neuronal excitation due to less inhibitory tone. Neural transmission is affected by synaptic autoantibodies, as are IgLON5 autoantibodies, which can result in increased tau deposits in cognition-relevant areas such as the hippocampal and entorhinal regions as demonstrated recently in a patient (Erro et al. 2019). The broader impairment of neuronal networks contributing to cognition can be assumed due to the ubiquitous brain localization of MOG antibodies, as these autoantibodies induce experimental autoimmune encephalitis (Wegener and Panzer 2020). The specific interaction between relevant brain regions in cognition [such as the cerebellum and limbic system termed limbic cerebellum (Schmahmann 2019)] might be impaired by the accumulation of specific autoantibodies such as ITPR1 antibodies known to induce a form of autoimmune cerebellar ataxia (Weihua et al. 2019).

### Markers of neurodegeneration and autoantibodies associated with cognitive impairment

Our findings show that Aß42 and the Aß42/40 ratio are pathogenic markers in Alzheimer’s disease, but not in cognitive impairment associated with autoantibodies, thus confirming prior knowledge. Autoantibody-associated cognitive decline did not lead to a ß-amyloidopathy-related neurodegenerative process. We were surprised to observe that total tau protein was not elevated in the ABS + COG group, although previous studies have reported elevated tau protein in patients suffering from antibody-positive autoimmune encephalitis (Constantinescu et al. 2016). As our ABS + COG subgroup only contains some patients with autoimmune encephalitis, these findings might indicate a distinction between autoantibody-associated isolated cognitive dysfunction and autoimmune encephalitis in their degree of secondary neurodegeneration. We detected no total tau protein elevation in the majority of ABS + COG patients, indicating no further neurodegenerative process like that observed in rapidly progressing Alzheimer’s disease in apoliprotein E (APOE) ε4-carriers (Wattmo et al. 2020).

### Pathogenic role of autoantibodies

The cognitive impairment in 9 of 26 (35%) of our patients tested for autoantibodies is likely caused by possible autoimmune encephalitis. In another 5 of 14 (36%) antibody-positive patients, we diagnosed a probable autoimmune-based psychiatric syndrome (for review see Hansen et al. 2020a) presenting as cognitive dysfunction. Only one patient presented no discernable autoimmune etiology. The pathological significance of serum autoantibodies in conjunction with no other indications of an underlying immunopathology (verified by additional diagnostics) is unclear. However, and although other hints indicating autoimmunity are present, the relevance of these serum autoantibodies is often incompletely understood. For example, autoantibodies might be an epiphenomenon not related to the main pathophysiology such as cellular immunity, i.e., cytotoxic CD8 + T-cells (Langenbruch et al. 2020); GAD65 autoantibodies themselves do not represent a major part of the autoimmunity process, as intrathecal GAD65 antibody production is often unproven (for review see Graus et al. 2020). Other autoantibodies, such as those against membrane surface antigens, often play a relevant role in immunopathology, such as NMDAR autoantibodies (Malviya et al. 2017). We thus need additional novel biological markers for those patients with cognitive dysfunction who do not reveal autoimmune indicators according to autoimmune encephalitis or autoimmune-based psychiatric syndromes guidelines (Graus et al. 2016; Hansen et al. 2020a). This point is further corroborated by the fact that serum autoantibodies alone might occur in healthy humans that do not reveal cognitive decline, as a study by Levin demonstrated (Levin et al. 2010). In addition, autoantibodies may not even contribute to the disease progress and pathophysiology, as they can play a protective role, i.e., specific autoantibodies in Alzheimer’s disease (Sim et al. 2020). Another relevant issue is that the presence of serum autoantibodies seems to depend on the blood–brain barrier’s integrity, as its breakdown is accompanied by the numerous and highly varied human brain reactive autoantibodies targeting membrane proteins (Levin et al. 2010). Thus, it is reasonable to presume a transient blood–brain barrier breakdown as a precondition in those patients with brain-reactive serum autoantibodies, although we discerned no relationship between the occurrence of blood–brain barrier disturbances and the albumin quotient in CSF and the degree of cognitive impairment. Taken together, it remains unclear whether there is a role and if so, what the pathogenic role is of various proven serum autoantibodies in patients. What this question raises, in particular, is whether other autoimmune indicators are lacking. CSF analysis including specific autoantibody analysis is, therefore, the best approach for later therapeutic decisions.

### Limitations

Our cohort is too small to draw any practicable conclusions. Furthermore, our cohort is heterogeneous, thus precluding conclusions on the autoantibody frequency of individual autoantibodies in cohorts presenting cognitive impairment. Furthermore, the frequency of autoantibodies detected in serum and CSF only enables us to estimate the potential frequency within our entire memory cohort, as we only tested those patients presenting possible autoimmunity due to indicators, and not all patients with cognitive impairment. Moreover, it is unclear whether these potential autoimmune indicators really do indicate those patients with possible underlying autoantibodies. Further systematic research is required in different groups suffering cognitive impairment to clarify this issue. The wide variability of the 11 autoantibodies we detected in 15 patients might argue for the plethora of autoantibodies involved in several types of cognitive dysfunction. But this might also imply that specific autoantibodies play no pathogenic role.

## Conclusions

Our study in a memory cohort examined how often autoantibodies can be discerned in patients with cognitive dysfunction revealing additional hints for autoimmunity. The fact that we detected autoantibodies in 58% of patients suffering cognitive impairment (among those whom we had screened for autoantibodies) suggests an underestimated phenomenon that memory clinic staffs should be aware to diagnose and treat patients promptly and adequately. Furthermore, we cannot exclude that the autoantibody frequency is even higher, given that we did not test for specific autoantibodies in all patients. Our results are promising for the future establishment of testing serum and CSF autoantibodies in patients with cognitive dysfunction and indicators of autoimmunity. In addition, as autoimmune indicators are detected in patients with and without antibodies as well as in those with Alzheimer’s disease, further research is required to help discover sensitive clinical and molecular biomarkers of autoimmunity besides autoantibodies as early possible indicators of autoimmune-related cognitive impairment.

## Data Availability

Data is available.

## Abbreviations

ABS + COG: Autoantibodies and cognitive impairment
ABS − COG: No autoantibodies and cognitive impairment
AD: Alzheimer’s disease
ADCOG: Alzheimer’s disease with cognitive impairment
AMPAR: α-Amino-3-hydroxy-5-methyl-4-isoxazolepropionic acid receptor
ARHGAP26: Rho GTPase-activating protein 26
Aß40: Beta Amyloid 40
Aß42: Beta Amyloid 42
CASPR2: Contactin-associated protein like 2
CERAD: Consortium to establish a registry for Alzheimer’s disease
CSF: Cerebrospinal fluid
DPPX: Dipeptidyl-peptidase-like protein 6
FTD: Frontotemporal dementia
GAD65: Glutamic acid decarboxylase 65
GDS: Geriatric depression scale
GluA3: Subunit ionotropic glutamate receptor 3
IgA: Immunoglobulin A
IgG: Immunoglobulin G
IgM: Immunoglobulin M
ITPR1: Inositol 1,4,5-triphosphate receptor 1
KCNA2: Potassium voltage-gated channel subfamily A member 2
LBD: Lewy Body dementia
MCI: Mild cognitive impairment
MMSE: Mini Mental Status Examination
MOG: Myelin oligodendrocyte glycoprotein (MOG)
MRI: Magnetic resonance imaging
N: Number
NMDAR: *N*-Methyl-d-aspartate receptor
OCB: Oligoclonal bands
PDD: Parkinson’s disease dementia
Pre-GLRA1: Pre glycine receptor alpha 1
pTau181: Phosphorylated tau protein 181
QAlb: Quotient albumin
QIgG: Quotient immunoglobulin G
QIgM: Quotient immunoglobulin M
SCD: Subjective cognitive decline
VD: Vascular disease
GABAAB1/2: γ-Amino butyric acid A/B receptor
